# Beyond the gut: a comprehensive meta-analysis on *Helicobacter pylori* infection and cardiovascular complications

**DOI:** 10.1186/s12941-025-00788-6

**Published:** 2025-03-18

**Authors:** Somayeh Yaslianifard, Fatemeh Sameni, Kimia Kazemi, Yousef Atefpour, Bahareh Hajikhani, Ali Baradaran Bagheri, Shahrooz Yazdani, Masoud Dadashi

**Affiliations:** 1 Department of Microbiology, School of Medicine, Alborz University of Medical Sciences, Karaj, Iran; 2 Department of Microbiology, Faculty of Medicine, Shahed University, Tehran, Iran; 3 Molecular Microbiology Research Center, Faculty of Medicine, Shahed University, Tehran, Iran; 4 Department of Microbiology, School of Medicine, Shahid Beheshti University of Medical Sciences, Tehran, Iran; 5 Department of Neurosurgery, Shahid Madani Hospital, Alborz University of Medical Sciences, Karaj, Iran; 6 Department of Cardiology, School of Medicine, Alborz University of Medical Sciences, Karaj, Iran; 7 Cardiovascular Research Center, Shahid Rajaei Hospital, Alborz University of Medical Sciences, Karaj, Iran; 8 Non-Communicable Diseases Research Center, Alborz University of Medical Sciences, Karaj, Iran

**Keywords:** *Helicobacter pylori*, Cardiovascular disease, Coronary artery disease, *cagA*

## Abstract

**Background:**

*Helicobacter pylori* (*H*. *pylori*) is known to induce chronic inflammatory conditions, and interactions between the host immune system and pathogen have diverted attention toward investigating its correlation with extra-gastrointestinal disorders.

**Objective:**

The present study aimed to assess the rate of *H. pylori* infection in cardiovascular disease (CVD) through a systematic review and meta-analysis.

**Methods:**

We conducted a large-scale meta-analysis to determine the prevalence rates of *H. pylori* infection in vascular diseases. Articles from PubMed/Medline, Web of Science, and Embase databases published between 2000 and 2023 were included for analysis. We used multiple independent observers to extract data, calculated the pooled frequency of *H. pylori* in vascular diseases using a random effect model, and reported the results as a weighted average based on the study population. The main outcome measures were presented with 95% confidence intervals (CI).

**Results:**

In 87 included studies, the prevalence of *H. pylori* infection in vascular diseases was 56.7% worldwide. 14.25% of *H. pylori* isolates harbored the *cagA* gene. The predominant vascular complication was coronary artery disease (CAD) (31.07%), primarily documented in Europe. This meta-analysis revealed a declining emphasis on studying the association of *H. pylori* infection with vascular disease in recent times.

**Conclusion:**

According to this meta-analysis, *H. pylori* infection has a high frequency in CVD and may increase the risk of vascular diseases. However, further research is required, particularly in nations with limited data.

## Introduction

Cardiovascular disease (CVD) is the leading cause of global mortality and morbidity [1]. The common risk factors of CVD are hypertension, old age, physical inactivity, diabetes mellitus, dyslipidemia, obesity, and smoking [2]. Moreover, inflammatory factors and oxidative stress are among the novel risk factors that may be useful for CVD prevention [3]. *Helicobacter pylori* *(H. pylori)* infection is a risk factor for developing coronary heart disease, arrhythmia, and acute myocardial infarction [4]. The inflammatory responses triggered by the *H. pylori* infection are the main underlying causes of cardiovascular complications [5]. *H. pylori* strains possess a cytotoxin-associated gene A (*cagA*) is more virulent and more strongly related to the risk of coronary atherosclerosis [6]. These strains increase the activity of endothelial cycloxygenase-1 and −2. Also, *cagA*-induced inflammation may promote the release of cytokines (including IL-8), tumor necrosis factor- α (TNF-α), and T and B lymphocytes, thereby causing cardiac diseases [5]. Particularly, an autoimmune reaction might be proposed that involves cross-reactivity between anti-*cagA* antibodies and vascular wall antigens, implying that these antibodies may contribute to the activation of inflammatory cells within atherosclerotic lesions. *H. pylori* carries the heat shock protein-60 (HSP60), which is identical to an arterial cell surface protein found in endothelial cells [7, 8]. Therefore, an immune response to *H. pylori* may induce immune cross-reaction between human and bacterial HSP60, which in turn leads to an autoimmune reaction and local inflammation of the artery.

Chronic inflammatory response. This gram-negative bacilli infection increases fibrinogen, blood leukocytes, and homocysteine levels, stimulates the release of C-reactive protein (CRP), induces hypercoagulability, and increases the production of proinflammatory inflammatory metabolites. An increase in cytokines (IL-1, IL-6, and IL-8) alters blood vessel motility and induces endothelial dysfunction, resulting in the beginning, progression, and consequences of atherosclerotic plaque formation, thus raising the risk of heart attack [9, 10].

Furthermore*, H. pylori* infection is linked to dyslipidemia. Pro-inflammatory cytokines, particularly TNF-α, can block lipoprotein lipase and increase free radical generation. Patients with *H. pylori* infection have lipid profile abnormalities, including low HDL cholesterol and high total cholesterol, low-density lipoprotein (LDL) cholesterol, and triglyceride levels. Early events in atherosclerosis include increased transcytosis of low-LDL across the endothelium and the oxidation of LDL deposited within the subendothelial region [11]. Furthermore, oxidised LDL stimulates IL-8 production, which is greatly increased by *H*. *pylori* infection, resulting in increased recruitment of T lymphocytes and smooth muscle cells, leading to atherosclerotic plaques. Studies have discovered bacterial DNA in atherosclerotic plaques, where it generates patches of infection that contribute to heart disease [12].

This systematic review and meta-analysis aimed to determine the worldwide prevalence of *H. pylori* infection and its association with CVD risk, given its importance.

## Methods

### Search strategy

A thorough, systematic search for appropriate papers published in PubMed/Medline, Web of Science, and Embase was done. All research published in English between 1998 and 2023 were reviewed.

The search approach included the following terms: “*Helicobacter pylori*” OR “*H. pylori*” AND “cardiovascular disease” OR “coronary heart disease” OR “coronary artery disease” OR “myocardial infarction” OR “ischemic heart disease” OR “atherosclerotic stroke” OR “CHD” OR “CVD” OR “CAD”. We utilized MeSH terms while searching PubMed/Medline and Embase. This method was independently examined by two distinct investigators (BH and FS). The PICO method was used to create the inclusion and exclusion criteria for study selection. Therefore, we assessed the data on P (Patient, Population, or Problem) = patients with CVD, I (Intervention or exposure) = *H. pylori* infection, and C ( Comparison) = not available, and O (Outcome) = Relationship between *H. pylori* infection and risk of CVD.

All clinical studies investigating the presence of *H. pylori* infection in patients with coronary artery disease (CAD) were included, except for articles that reported only the prevalence of *H. pylori* or the prevalence of CAD alone, duplicated articles, abstracts presented at conferences, reviews, book chapters, case reports, case series, and meta-analyses. Relevant prevalence studies were considered. In the following phase, two investigators (KK, YA) reviewed the titles and abstracts of all selected publications.

### Data extraction

The first author's name, the year of publication, the type of study, the nation where the study was done, the age and gender of the patients, the number of patients with CAD, the number of patients *H. pylori*, and the type of CAD were extracted from all eligible publications and entered into a data extraction form. To eliminate bias, two writers separately recorded the data (SY, SHY). The disagreement was addressed by discussion amongst the authors (MD, AB).

### Quality assessment

The critical appraisal checklist provided by the Joanna Briggs Institute (JBI) was used to perform a quality assessment of the studies [13].

### Statistical analyses

Statistical analyses were conducted using Comprehensive meta-analysis (CMA) software (version 2.0, Biostat, USA). The pooled frequency with 95% confidence intervals (CI) was calculated using the random effect model. Cochran’s Q and the I2 statistic were used to analyse heterogeneity between studies. To investigate heterogeneity, subgroup analyses stratified by disease type were conducted. Begg's test was used to examine publication bias statistically (a P value of less than 0.05 indicated statistically significant publication bias).

## Results

### Characteristics of included studies

**Fig. 1:**
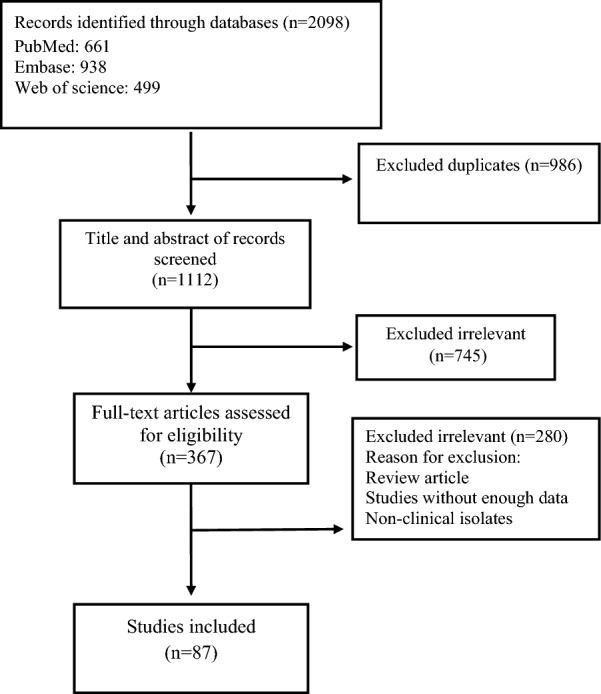
Flow chart of study selection for inclusion in the systematic review

**Fig. 2:**
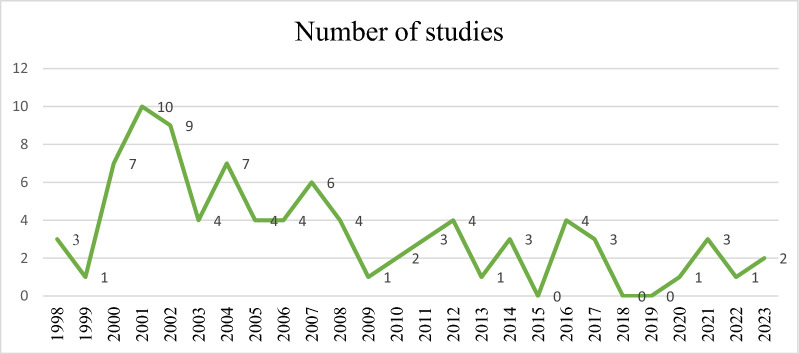
Global CVD rates among patients with *H. pylori* during 1998–2023

Overall, 2098 citations were found during the first database searches. Our data was gathered from three databases, and some duplicate research were included. Following the removal of 986 duplicates, there were 1112 non-duplicate studies. After reviewing the titles and abstracts, we selected 745 studies that were not relevant. In addition, 280 irrelevant items were removed throughout the full-text screening process. The final analysis included 87 studies (see Table 1). Figure 1 depicts the rationale for eliminating papers at various levels of the evaluation. According to published sources, males outnumbered females by 6.7 times. Table 2 shows that the majority of the studies were published between 1998 and 2006 (56.3%), 2007 to 2015 (27.6%), and 2016 to 2023 (16.1%). Most of the articles included in the present study were published in Europe (60.9%), and Italy had the most reported articles in this continent (28.3%). Figure 2 shows the number of articles published each year.

**Table 1:** Characteristics of the included studies

First author	Published time	Country	Number of patients	Number of *H. pylori* isolates	Mean age	Male	Female
Tano [22]	2000	Italy	206	89	58 ± 7	196	10
Garcia [23]	2001	Spain	100	75	NR	75	25
Pinar [24]	2004	Turkey	33	14	NR	NR	NR
Sheehan [25]	2005	Ireland	227	139	59.4	162	65
Jeong [26]	2003	Korea	272	171	59.1 ± 10.4	208	64
Ziver [27]	2010	Turkey	96	67	62.94	76	20
Grub [28]	2012	Norway	119	51	NR	76	43
Lanza [29]	2004	Italy	104	53	NR	53	51
Preusch [30]	2004	Germany	190	104	59.5 ± 11.7	125	65
Sawayama1 [31]	2005	Japan	62	48	71.5 ± 11.3	40	22
Kaehler [32]	2006	Germany	205	100	NR	168	37
Bai [33]	2017	Pakistan	109	82	50.93 ± 8.13	55	54
Davoudi [34]	2011	Iran	69	40	60.5 ± 1.05	53	16
Masoud [35]	2005	Iran	91	59	64.3 ± 10	48	43
Heuschmann [36]	2001	Germany	145	67	74.6	68	77
Fan [37]	2021	China	76	31	70.1	50	26
Palm [38]	2016	Germany	470	182	NR	282	188
Vijayvergiya [39]	2006	India	90	38	NR	NR	NR
Vafaeimanesh [40]	2014	Iran	62	45	NR	36	26
Ponzetto [41]	2002	Italy	80	64	56.7	58	22
Shmuely [42]	2014	Israel	173	110	68.5	124	49
Gabrielli [43]	2004	Italy	105	75	68 ± 8	49	56
Bloemenkamp [44]	2002	Netherlands	228	91	48.0 ± 7.0	0	228
Yamamoto [45]	2012	Japan	64	33	72.2 ± 7.5	38	26
Yang [46]	2011	China	150	101	66 ± 9	105	45
Sawayama2 [47]	2008	japan	69	55	63.38 ± 10.28	33	36
Padmavati [48]	2012	India	110	69	NR	NR	NR
Nikolopoulou [49]	2008	Greece	288	146	62.4 ± 0.61	258	130
Vahdat [50]	2007	Iran	222	149	NR	NR	NR
Niccoli1 [51]	2010	Italy	40	24	63.3 ± 11	33	7
Witherell [52]	2003	USA	121	84	55	68	53
Figura1 [53]	2014	Italy	103	41	65 ± 8	70	33
Hoffmeister [54]	2001	Germany	238	90	NR	NR	NR
Schiele [55]	2001	France	180	61	56	158	22
Li [56]	2021	China	88	54	NR	28	60
Niccoli2 [57]	2017	Italy	181	108	64 ± 13	155	26
Badran [58]	2007	Egypt	185	113	NR	111	74
Pietroiusti [59]	2002	Italy	199	137	NR	119	80
Dore [60]	2003	Italy	32	23	69 ± 8.3	27	5
Kilic [61]	2006	Turkey	29	14	37.6	23	7
Iriz [62]	2008	Turkey	42	11	57.3 ± 11.4	33	9
Ameriso [63]	2001	Argentina	38	20	67 ± 9	29	9
Kowalski1 [64]	2002	Germany	46	22	62.7 ± 9.17	37	9
Assanelli [65]	2004	Italy	48	29	35.4	43	5
Schumacher [66]	2002	Norway	193	86	55	158	35
Yusuf [67]	2002	England	40	19	81	NR	NR
Choussat [68]	2000	France	79	33	NR	50	29
Stone [69]	2002	England	310	157	NR	NR	NR
Elizalde [70]	2004	Spain	92	49	NR	NR	NR
Oijen [71]	2007	Netherlands	376	186	64.7	227	149
Horne [72]	2002	USA	415	216	62 ± 11	332	83
Kahan [73]	2000	Sweden	99	67	66.4	76	23
Kowalski2 [74]	2001	Germany	96	67	NR	NR	NR
Aceti [75]	2004	Italy	40	28	60.67 ± 12.42	34	6
Aldhalmi [76]	2020	Iraq	100	56	NR	50	50
Vcev [77]	2007	Croatia	90	71	49.2	61	30
Osawa [78]	2001	Japan	206	137	59.8 ± 0.5	175	31
Fallah [79]	2016	Iran	96	77	51.32 ± 2.61	68	28
Jukic [80]	2017	Croatia	150	87	62.61 ± 10.23	109	41
Park [81]	2006	Korea	125	100	66.74 ± 7.69	63	62
Grau [82]	2001	Germany	109	57	60 ± 14.7	73	36
Moayyedi [83]	2003	England	467	274	70.5	239	228
Lazaraki [84]	2008	Greece	102	57	75.44 ± 19	48	54
Sarraf [85]	2001	Iran	103	49	55 ± 8.0	80	23
Kanbay [86]	2005	Turkey	151	91	48.1	93	58
Tabata [87]	2016	Japan	253	112	NR	NR	NR
Figura2 [88]	2002	Italy	63	50	65	NR	NR
Galante [89]	2000	Italy	63	32	64.1 ± 9.34	47	16
Murray [90]	2000	England	259	183	NR	74	185
Rogha [91]	2012	Iran	62	30	62.4 ± 9.5	42	20
Kowalski3 [92]	2001	Germany	100	81	54	52	48
Franceschi [93]	2013	Italy	54	23	44 ± 17	40	14
Tsai [94]	2000	Taiwan	165	114	65.5 ± 8.6	113	52
Bonaventura[95]	2007	Italy	58	34	62.8 ± 9.6	NR	NR
Gunn [96]	2000	England	342	206	65.1 ± 11.9	229	113
Jin [97]	2007	Korea	175	71	62.6 ± 8.6	111	64
Assadi [98]	2009	Iran	30	15	53.20 ± 6.16	12	18
Azarkar [99]	2011	Iran	73	42	59.8 ± 11.5	53	20
Khurshid [100]	1998	USA	119	55	NR	NR	NR
Koenig [101]	1999	Germany	312	126	57.7 ± 7.4	267	45
Darvishi [102]	2016	Iran	84	51	63.12 ± 13.70	41	NR
Markus [103]	1998	England	238	140	65.9	41	43
Regnstrom [104]	1998	Sweden	92	39	40.9	92	0
Warme1 [105]	2023	Sweden	198	39	60	56	142
Warme2 [106]	2020	Sweden	289	57	67	222	67
Alfy [107]	2023	Egypt	100	72	58 ± 12	66	34
Azeem [108]	2022	Egypt	100	60	NR	56	44

NR: Not reported

**Table 2:** Prevalence of published studies reporting *H. pylori* in CVD in different time periods worldwide

Continent	1998–2006 No. of studies	2007–2015 No. of studies	2016–2023 No. of studies
Africa (3)	NR	1	2
Asia (27)	8	12	7
America (4)	4	NR	NR
Europe (53)	37	11	5
Oceania	NR	NR	NR
Total (87)	49	24	14

	Country (N)	Country (N)	Country (N)
Africa (No, %)			
Egypt (3, 100%)	NR	1	2
America (No, %)			
USA (3, 75%)	3	NR	NR
Argentina (1, 25%)	1	NR	NR
Asia (No, %)			
Iran (10,37.03)	2	6	2
Iraq (1, 3.7)	NR	NR	1
China (3,11.11)	NR	1	2
Japan (5, 18.51)	2	2	1
India (2, 7.4)	1	1	NR
Pakistan (1,3.7)	NR	NR	1
Israel (1, 3.7)	NR	1	NR
Taiwan (1,3.7)	1	NR	NR
Korea (3, 11.11)	2	1	NR
Europe			
Netherlands (2,3.77)	1	1	NR
Spain (2, 3.77)	2	NR	NR
Italy (15,28.30)	10	4	1
France (2,3.77)	2	NR	NR
Sweden (4,7.54)	2	NR	2
Greece (2, 3.77)	NR	2	NR
Norway (2, 3.77)	1	1	NR
Croatia (2, 3.77)	NR	1	1
Ireland (1,1.88)	1	NR	NR
Germany (10,18.86)	9	NR	1
Turkey (5,9.43)	3	2	NR
England (6,11.32)	6	NR	NR

NR: Not Reported

### Prevalence of *H. pylori* infection in CVD

**Fig. 3:**
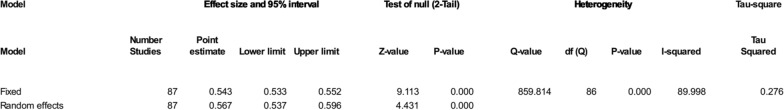
Frequency of *H. pylori* among patients with CVD worldwide

**Fig. 4:**
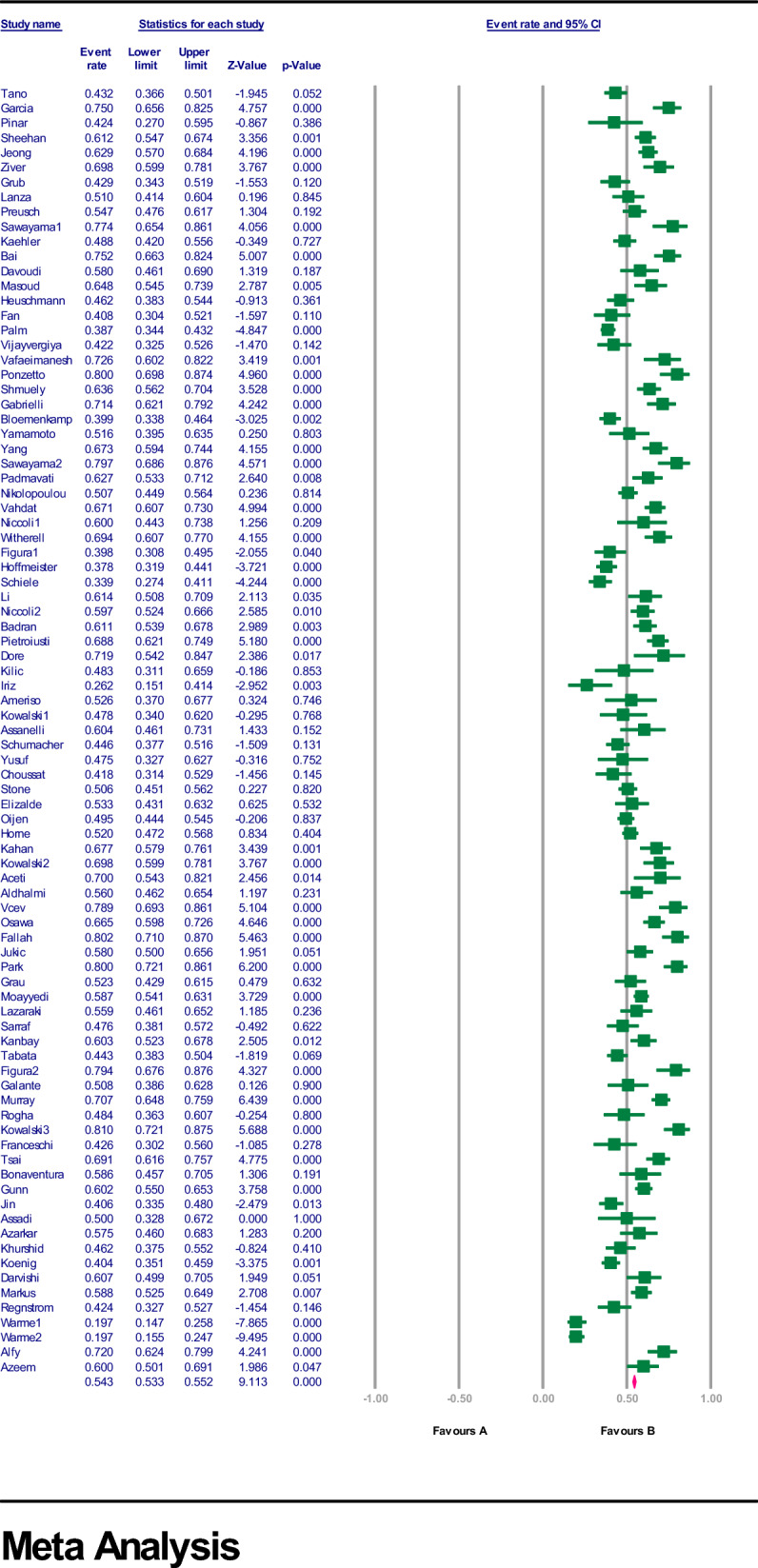
forest plot of the meta-analysis on the prevalence of *H. pylori* among patients with CVD worldwide

**Fig. 5:**
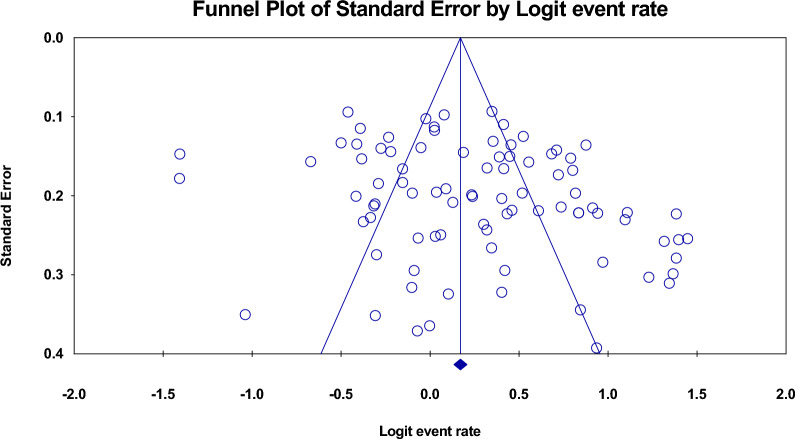
funnel plot of the meta-analysis on the prevalence of *H. pylori* among patients with CVD worldwide

**Fig. 6:**
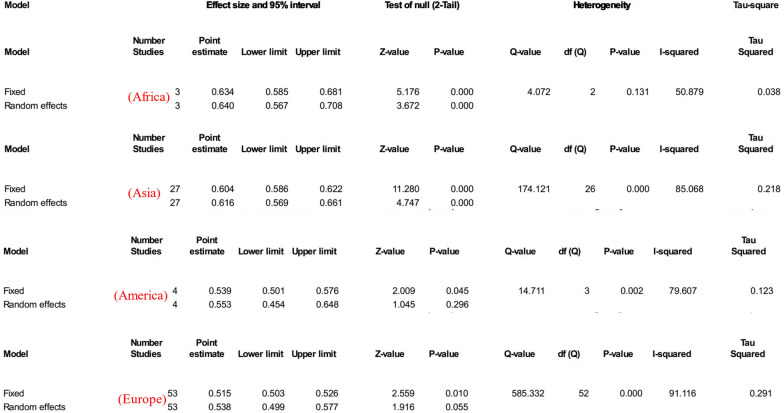
Frequency of *H. pylori* among patients with CVD in different continents

**Fig. 7:**
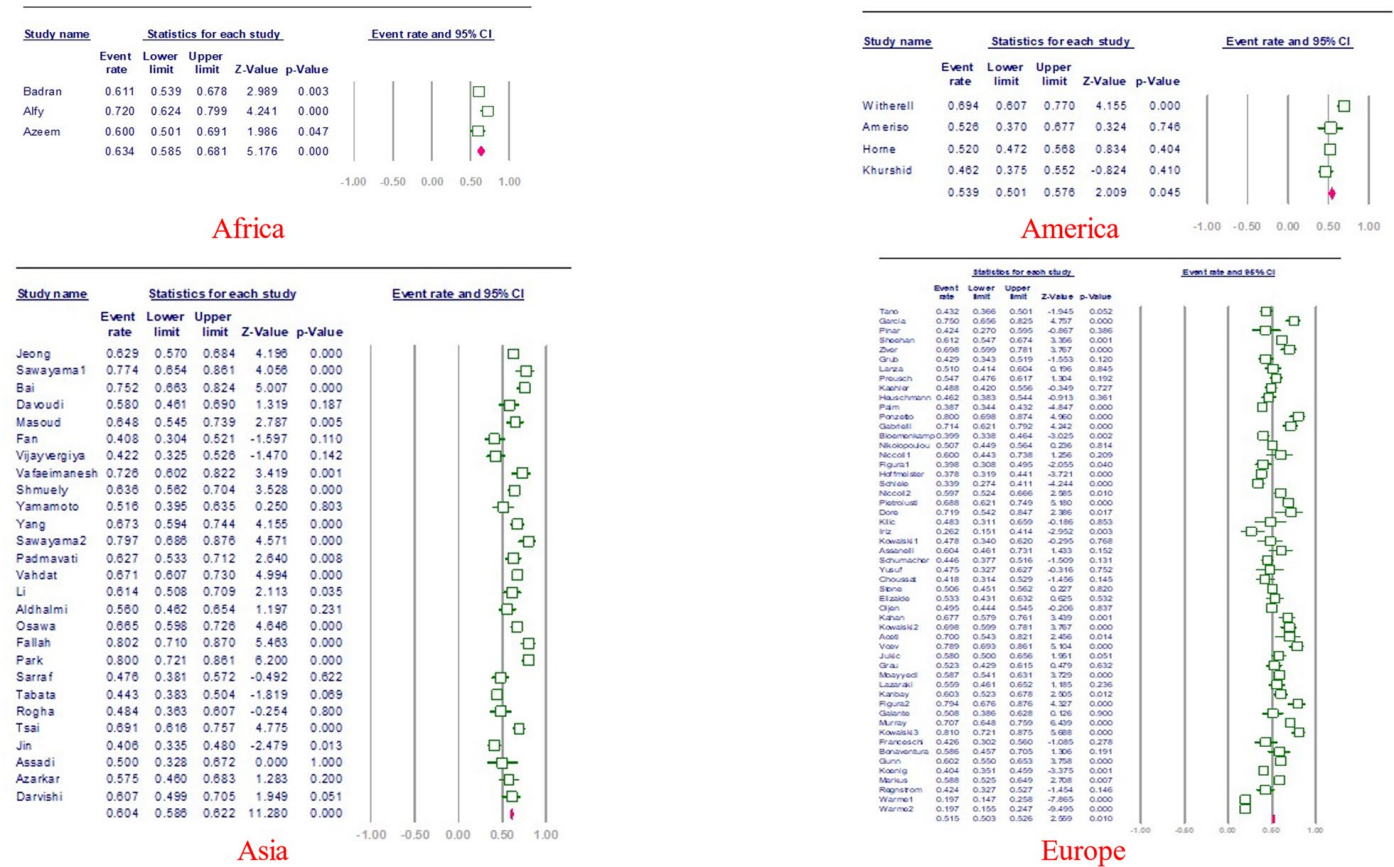
forest plot of the meta-analysis on the prevalence of *H. pylori* among patients with CVD in different continents

**Fig. 8:**
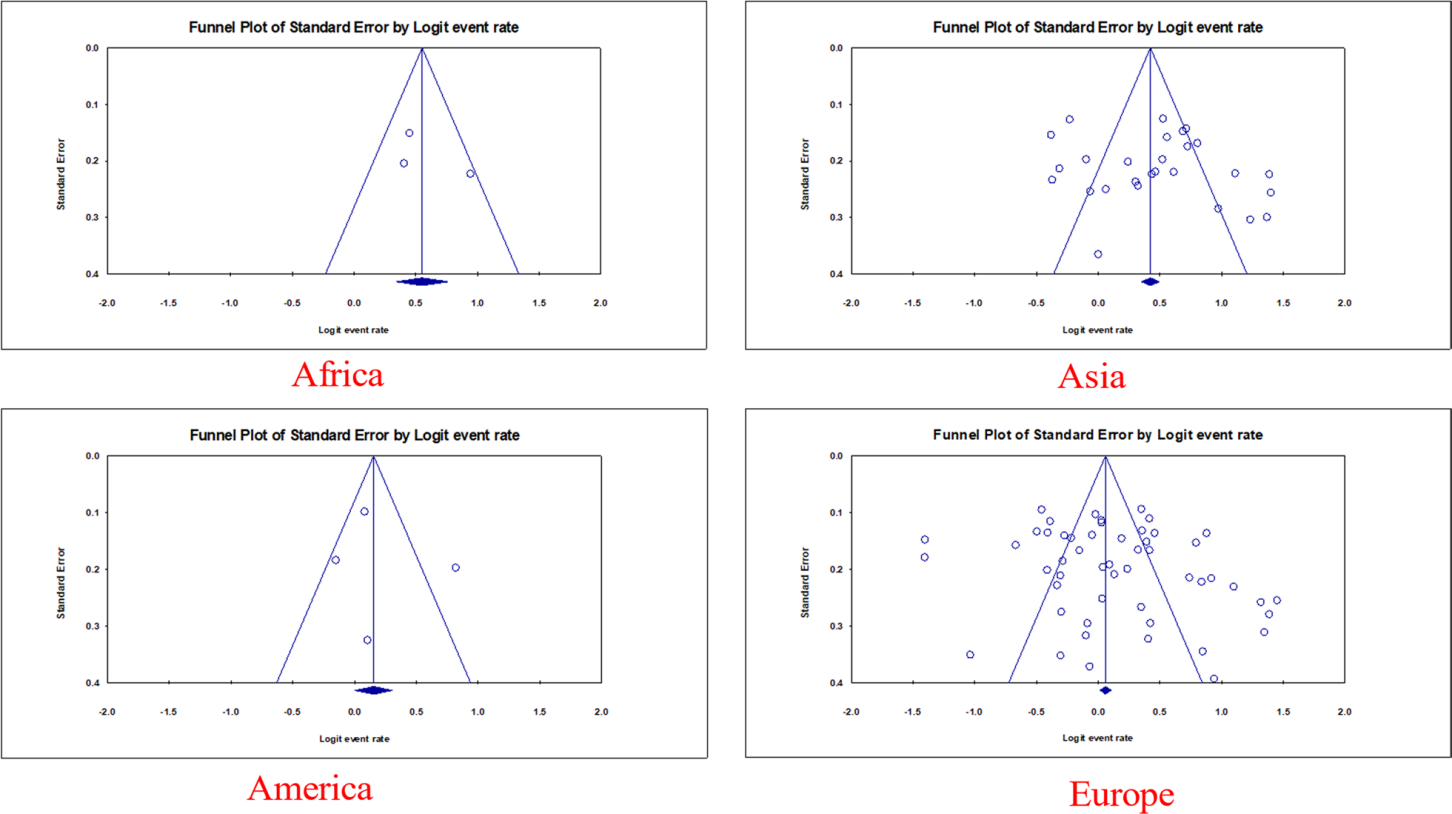
funnel plot of the meta-analysis on the prevalence of *H. pylori* among patients with CVD in different continents

The estimated rate of *H. pylori* in individuals with CVD was 56.7% [95% confidence interval (CI) 53.7–59.6, I2: 89.9%]. (Fig. 3). Figures 4 and 5 show the forest and funnel plots, respectively. Africa had the greatest frequency of *H. pylori* infection in CVD (64%), followed by Asia (61.6%), America (55.3%), and Europe (53.8%) (Figs. 6–8), there were no reports from Oceania.

### Prevalence of *H. pylori cagA* gene in patients with vascular diseases

As indicated in Table 3, 14.25% of the 6775 *H. pylori* isolates had the *cagA* gene. The highest prevalence of this gene was seen in Europe (87.1%), with the highest frequencies found in Italy (34.7%) and England (32.8%). None of the *H. pylori* isolates from America included the *cagA* gene.

**Table 3:** Distribution of *cagA* gene among *H. pylori* in vascular diseases in different continent

Continent Country	n/N	n/N	country	N (%)
Africa (92)	92/6775 (1.35)	92/966 (9.5)	Egypt	92 (100)
Asia (33)	33/6775 (0.48)	33/966 (3.4)	China	33 (100)
America (0)	NR	NR	NR	NR
Europe (841)	841/6775 (12.41)	841/966 (87.1)	England	276 (32.8)
			Turkey	64 (7.6)
			Sweden	18 (2.2)
			Greece	12 (1.4)
			Italy	292 (34.7)
			Germany	179 (21.3)
Total (966)	966/6775(14.25)			

n: Number of *cagA* + isolates; N: Number of *H. pylori* positive isolates

### The most prevalent vascular disease among patients with *H. pylori* in different continents

The most common CVD manifestations in 6775 individuals with *H. pylori* infection were CAD (31.07%) and atherosclerotic stroke (22.45%), respectively. CAD was most common in Europe (43.75%), Asia (38%), Africa (5.3%), and America (2.87%). In Europe, CAD is mostly documented in Germany, Croatia, and Greece. According to published studies, atherosclerosis stroke occurred exclusively in Europe (17.07%) and Asia (5.37%). Cerebral microbleeds were the least common CVD documented in Asia (0.45%) (Table 4).

**Table 4:** Type of vascular diseases in *H. pylori* positive patients

Variables (No. of studies)	No. of patients in continents	No. of patients in countries	No. of patients /total (103)
Atherosclerotic stroke (15)	Europe (1157)	Germany (410)	1521/6775 (22.45)
		England (414)	
		Italy (276)	
		Greece (57)	
	Asia (364)	Iran (59)	
		Iraq (56)	
		Japan (48)	
		China (101)	
		Korea (100)	
Cerebral infarction (1)	Asia (33)	Japan (33)	33/6775 (0.48)
Cerebral microbleeds (1)	Asia (31)	China (31)	31/6775 (0.45)
CHD (8)	Asia (218)	Iran (81)	734/6775 (10.83)
		Japan (137)	
	Europe (516)	Italy (28)	
		Norway (86)	
		Germany (216)	
		Netherlands (186)	
Aortic aneurysm (1)	Europe (67)	Turkey (67)	67/6775 (0.98)
CAD (29)	Asia (800) 38.00	Iran (324)	2105/6775 (31.07)
		India (38)	
		Korea (242)	
		Israel (110)	
		Taiwan (86)	
	Africa (113)	Egypt (113)	
	Europe (921)	Italy (90)	
		Germany (270)	
		France (33)	
		Sweden (39)	
		Turkey (102)	
		Croatia (158)	
		Norway (51)	
		Spain (32)	
		Greece (146)	
	America (271)	USA (271)	
Acute coronary syndrome (6)	Asia (112)	Japan (112)	608/6775 (8.97)
	Europe (496)	Ireland (139)	
		England (157)	
		Italy (108)	
		Spain (92)	
Myocardial Infarction (15)	Asia (257)	Taiwan (28)	1115/6775 (16.45)
		Iran (78)	
		Pakistan (82)	
		India (69)	
	Europe (702)	Sweden (163)	
		Italy (150)	
		England (389)	
	America (84)	USA (84)	
	Africa (72)	Egypt (72)	
Cardiac syndrome X (2)	Asia (15)	Iran (15)	43/6775 (0.63)
	Europe (28)	Italy (28)	
Ischemic Heart Disease (3)	Europe (103)	Italy (84)	103/6775 (1.52)
		England (19)	
Stent implantation in a native coronary artery (1)	Europe (61)	France (61)	61/6775 (0.90)
Peripheral arterial disease (2)	Asia (55)	Japan (55)	146/6775 (2.15)
	Europe (91)	Netherlands (91)	
Idiopathic Dysrhythmias (1)	Europe (23)	Italy (23)	23/6775 (0.33)
Atherosclerosis (6)	Europe (51)	Turkey (28)	185/6775 (2.73)
		Italy (23)	
	Asia (54)	China (54)	
	America (20)	Argentina (20)	
	Africa (60)	Egypt (60)	

CHD: Coronary heart disease, CAD: Coronary Artery Disease

### The most prevalent clinical source, detection methods, and underlying diseases among patients with *H. pylori*

According to published studies, *H. pylori* is mostly isolated from the serum of patients with CVD (86.77%). Following that, there was a breathing test and a gastrointestinal biopsy (7.97% and 3.64%, respectively). *H. pylori* was identified mostly by serological techniques (86.77%). The stool antigen test was shown to be the least commonly utilised approach for identifying *H. pylori* (0.41%). In addition, the most common underlying illnesses among patients infected with *H. pylori* were hypertension (4.8%), diabetes (2.58%), and obesity (0.13%) (Table 5).

**Table 5:** Characteristics of clinical source, detection methods and underlying diseases of the included studies

	Variables (No of studies)	No. of studies	No. of patients	No. of patients/Total
Clinical source	Serum	67	5879	5879/6775 (86.77)
	Gastric biopsy	4	247	247/6775 (3.64)
	Stool	1	28	28/6775 (0.41)
	Breath	10	540	540/6775 (7.97)
	Tissue	5	81	81/6775 (1.19)
*H. pylori* detection method	Histology (H&E statining OR Giemsa staining)	2	126	126/6775 (1.86)
	serology	67	5879	5879/6775 (86.77)
	PCR	5	81	81/6775 (1.19)
	urease breath test	10	540	540/6775 (7.97)
	stool antigen test	1	28	28/6775 (0.41)
	Rapid urease test	2	121	121/6775 (1.78)
Underlying disease	Hypertension	9	331	331/6775 (4.8)
	Diabetes	8	175	175/6775 (2.58)
	Obesity	1	9	9/6775 (0.13)
	NR	77	6267	6267/6775 (92.5)

NR: Not Reported; H&E staining: Hematoxylin and eosin staining; PCR: Polymerase chain reaction; ELISA: Enzyme-linked immunosorbent assay

## Discussion

*H. pylori* has been reported to contribute to the development of CVD in a variety of ways, including causing inflammation, endothelial dysfunction, dyslipidemia, iron and vitamin B12 malabsorption, and elevating CRP levels [5]. Recent research have shown conflicting findings about the involvement of this bacterium in the development of vascular disorders. As a result, our study intends to offer precise statistics on the prevalence of *H. pylori* infection among patients with vascular disorders worldwide. In our analysis, the global prevalence of *H. pylori* infection among CVDs was 56.7%.

Europe has the highest incidence, followed by Asia, America, and Africa. It is worth noting that the considerable number of research conducted in Asia (31.03%) and Europe (60.92%), as opposed to America (4.59%) and Africa (3.44%), may have influenced these findings.

According to a meta-analysis published by Hooi et al., Africa has the highest incidence of *H. pylori* infection worldwide, whereas our findings show that the prevalence of *H. pylori* in individuals with vascular disease is the lowest worldwide. This issue may be attributable to the small number of studies undertaken on this continent [14].

Furthermore, the lack of eligible studies from Oceania means that there is a lack of access to prevalence statistics in this region.

Interestingly, England had the highest prevalence rate in Europe. It is important to note that all relevant studies in this country were done between 1998 and 2006. As a result, the figures obtained may not precisely represent the current prevalence rate in this country. Argentina has the lowest prevalence rate in Europe, which could be attributed to the country’s small sample size and limited number of research (see Figs. 7, 8).

*CagA* is a surface protein that is associated with a more virulent strain of *H. pylori* [15]. According to Huang’s study infection with *cagA* positive strain leads to an increase in the risk of atherosclerosis [16]. In our investigation, the *cagA* gene was not commonly found in *H. pylori* isolates. This could be because many research have not looked at the presence of this gene. There has been no record of the *cagA* gene in America, hence there are no statistics on *H. pylori cagA* positive strains on this continent. The high incidence of *H. pylori cagA* positive strain in Europe may be linked to the availability of study undertaken on this topic in this region, whereas other places have minimal data available, since 90% of the studies that offered *cagA* statistics are related to the European countries.

Our findings demonstrated the most common type of CVD in *H. pylori* patients was CAD and mainly reported in Europe. Daponte-Codina et al. reported that CAD accounts for 20% of all mortality in Europe and is the most common form of CVD [17]. The high number of reports on CAD in the world is proof of the claim that *H. pylori* plays a significant role in causing this class of CVD. *H. pylori*, with the help of *cagA* and the type IV secretion system, causes hyperhomocysteinemia caused by malabsorption and incomplete metabolism of folate and vitamin B12, as well as molecular mimicry, leading to major changes in the lipid profile of coronary arteries and the development of CAD [5].

Atherosclerotic stroke was the second CVD diagnosed in *H. pylori* patients. The disease was most prevalent throughout Europe and England. Given the high number of heart attacks in England, the identification, treatment, and eradication of *H. pylori* can be employed as a reasonable and cost-effective method to avoid atherosclerotic strokes and myocardial infarction [6]. The lowest rate of Atherosclerotic stroke was reported in Japan. The results suggested that Japanese-style dietary patterns and characteristic Japanese food intake would contribute to reducing CVD risk [18].

Furthermore, we explored cerebrovascular disorders in the current study. Unfortunately, because of the complexity of sampling and the scarcity of samples, these disorders have far less literature than CAD. As a result, the conclusions and figures derived from their study are confusing. As a result, only two research met the criteria for inclusion in our study. Both studies were from Asia, one from Japan with 0.48% of all instances of cerebral infarction and the other from China with 0.45% of all cases of cerebral microbleeds. These findings revealed that researchers from other continents have focused on the potential link between *H. pylori* infection and cerebrovascular disease [7].

Serological approaches were the most commonly employed diagnostic tools in our investigation. Because of the requirement for upper endoscopy and the invasive nature of these procedures, their usage has been limited. It is crucial to highlight that utilising serological tests to diagnose *H. pylori* infection can have an impact on the accuracy of the results because these tests have limits in discriminating between early and late infections. According to Bordin's study, the noninvasive gold standard for detecting *H. pylori* infection is the urea breath test, which has a sensitivity of 96–100% and a specificity of 93–100%. In contrast, the serological test for this illness has a sensitivity of 85% and a specificity of 79% [19].

In the articles included in our study, underlying diseases have been examined as risk factors for cardiovascular diseases. Our findings demonstrated that hypertension is the most frequently occurring underlying disease among patients.

A study by Fang et al. found a significant link between *H. pylori* and hypertension, a key risk factor for cardiovascular disease [20]. It is important to emphasise that the low prevalence rate of hypertension in the current study can be related to the failure to examine underlying disorders in many of the selected papers. In the current investigation, *H. pylori* in CVD patients is primarily isolated from men. The likely reason for this is that the incidence of CVD in women is usually lower than in men. However, women have a higher mortality and worse prognosis after acute cardiovascular events [21].

Another remarkable feature is the 3.5-fold decrease in the number of reports on *H. pylori* in CVD worldwide between 2016 and 2023 compared to 1998–2006. Asia had the most reports in the last seven years. However, compared to 1998–2006 and 2007–2015, the number of published studies has decreased. The main thing regarding Europe is that the number of studies between 2016 and 2023 has declined by more than seven times when compared to 1998 to 2006. In the United States, reports of *H. pylori* in CVD were first published between 1998 and 2006. Another noteworthy discovery was that the number of publications released between 2016 and 2023 increased solely in Africa in contrast to 2007–2015. Moreover, with no publications emerging from Oceania, evaluating the progression of *H. pylori* infection in CVD over time in this region remains unfeasible. This study has certain drawbacks, which are as follows: First, since the frequency of the *cagA* gene was not explored in many researches, we could not identify a link between the presence of this gene and various forms of CVD. Second, there is minimal evidence on the relationship between *H. pylori* infection and underlying illnesses in CVD patients. As a result, we cannot assess the link between *H. pylori* infection and the development of underlying disorders. Third, a paucity of studies in diverse geographic regions, such as Oceania and Africa, limits the capacity of researchers to collect precise figures on the prevalence of *H. pylori* among CVD patients worldwide.

## Conclusion

Our findings revealed a high frequency of *H. pylori* infection among CVD patients, as well as the potential link between *H. pylori* infection and an elevated risk of cardiovascular problems. *H. pylori* infection is most commonly associated with CAD and least associated with idiopathic dysrhythmias. Additionally, serological tests are the most commonly employed to identify *H. pylori*, with the usage of more beneficial procedures such as the urease breath test and stool Ag test being limited. Moreover, further study is needed to investigate the effect of *H. pylori* infection in less well-studied kinds of CVD, such as cerebrovascular disorders. Eventually, more research is needed to determine the true prevalence rate of *H. pylori* in CVD, especially in areas where statistics are unavailable.

## Data Availability

No datasets were generated or analysed during the current study.
